# Nerve-Storms

**Published:** 1888-04-07

**Authors:** 


### The Doctor's Pulpit.

### NERVE-STORMS.

At different periods the world has different ways of regarding the same things, and expresses its judgments concerning them in different formulæ. What our grandfathers would have called a "towering rage" we designate a "nerve-storm"; and symptoms for which our grandmothers would have considered a sound whipping a proper measure, some of their descendants treat by a mild course of bromide of potassium. The martinet of stern temperament and limited culture is sure our forefathers were right; the better instructed and milder-mannered Galen of to-day inclines to the gentler discipline of his own times.

It is often said there is always truth on both sides; but that is not so. Wiseacres tell us the middle course is always the safest. That too is fallacious. There is *generally* truth on both sides, and the middle course is frequently the safest—but not always. In this particular instance, however, the common proverbial philosophy is entirely right, and there is really "much to be said for both parties."

What is a nerve-storm? Like other storms, it is a thing of infinite variety. A man in a frenzy of rage is carried away by a nerve-storm, and so too is a woman in a passion of grief. Any violent excitement or irrational worry, which is either uncontrollable, or only controllable by an effort that is almost superhuman, may be designated a nerve-storm.

Some questions proper to be asked are these: What are the causes of nerve-storms? Are such storms purely reflex phenomena? Are they capable of being curbed, managed, or entirely prevented by the subjects of them? What is the degree of responsibility attaching to the woman who is always losing her temper, or to the man who is for ever flying into a rage?

Here we enter a region where physics and metaphysics are difficult to distinguish, where mind and matter seem to be almost hopelessly intermixed. Many years ago the writer was passing the house of a coal miner named Ramsden. On a heap of cinders in front of the door a child of two was playing, and covering itself from head to foot with dust and dirt. The miner's wife, looking up from her work in the house, saw the child diligently frustrating all her efforts to keep it clean. In a moment, like a wolf or a tigress, she sprang to the door, seized the unlucky infant by the hair of its head, and lifting it bodily from the ground, began to cuff and kick it with every manifestation of uncontrollable rage. The tones of a remonstrating voice made her look round, and, seeing a spectator, she as hastily dropped the screaming denizen of the cinder heap, and rushing into the house, banged to the door in a transport of shame. There was a nerve-storm: there were, indeed, two nerve-storms—the one brought to a sudden termination by the prompt rising of the other.

What is the scientific explanation of such phenomena? The old-fashioned and, in this case, true explanation would have been that the woman was a wild, undisciplined creature who had too many children and too much work, and whose domestic kingdom was therefore always more or less in a condition of bad government and anarchy. She was a queen, whose divine right it was to reign; only she was a very incapable and very injurious monarch. But there is a modern explanation which is equally true, and which does not contradict, but only supplements the former. Mrs. Ramsden's rage, and Mrs. Ramsden's shame, were alike reflex manifestations. The image of the offending infant fell upon her eye; the impression produced on the retina was conveyed to the cerebrum, and by it passed in a moment down the spinal cord to the nerves and muscles of the legs and arms, and thus was completed a chain of cause and effect, strikingly simple, but illustrating some of the profoundest facts in the history of the human nervous system. The sight of a spectator produced a series of results exactly similar in every respect, although, of course, not identical.

The physiological probability is that the seeing of the child *in flagrante delicto* caused a sudden shock to the cerebrum, that what are called the vaso-motor nerves were at once temporarily paralysed by it, that consequently the cerebral blood-vessels became gorged in a moment, and that there was brought about a rapid production and discharge of nerve energy that resulted in all the manifestations which were so instructive, so grievous, and at the same time so grotesque.

What is the use of an explanation of this kind? It has these important uses: It teaches us how to measure conduct; how to apportion blame. It warns us of the danger of nerve-storms; and impresses upon us the necessity of controlling them in cases where control is possible. The cases where control is impossible are those of lunatics.

The poor woman whose strange behaviour, so long forgotten, has furnished an illustration for this brief physiologico-moral sermon, might easily have killed her child, although it is probable she loved it in her own way as much as the tenderest mother in the land. Many a murder is so committed. Not long ago an African chief, whose harem numbered hundreds, flew into a rage with his favourite wife and had her decapitated and her body thrown into the river on the instant. In less than an hour his grief was terrible; he refused his food, and for days and weeks was in a state bordering on distraction. His nerve-storm had destroyed his happiness for ever.

It does not require a philosopher to say that the unhappy chief, though a murderer, was a very different murderer from such a man as Palmer of Rugeley or Pritchard of Glasgow who deliberately and of set purpose took away their victim's life by poison. Such scoundrels as those deserved to be hanged ten times over, if that would have added to their punishment. But did the African chief really deserve to be hanged at all? We are not extenuating murder, or pleading that he who kills another in a fit of rage should not be punished—far from it. That would be to put a premium on undisciplined temper, and to encourage the world to prepare for universal restraint. But we do say that whenever such cases occur, and are dealt with by legal tribunals, they should be dealt with on lines entirely and absolutely different from those to which we are accustomed in cases of deliberate killing for some base ulterior purpose.

The closing word we have to say is a commonplace. Whatever be the physiological explanation of nerve storms, every sane and healthy person has it in his power to keep his own nerve-storm elements in perfect control. He who does not keep them in subjection must be held responsible for the actions he commits when the storm is raging. The thing to do is to avoid the beginnings of an outbreak, and this is best effected by acquiring and maintaining the habit of self-discipline and self-restraint. Every reasonable man keeps his own strait waistcoat in his own brain. With children it is not always well to substitute the bromide of potassium for the whipping or its equivalent. It may sometimes be necessary, and we do not agree with the rough-and-ready method of slapping a child whenever it cries without seeking to find out the reason. Children should be treated as justly, as candidly, and as intelligently as grown-up people. Their minds should always be satisfied that what is done to them is strictly right, just, and wise. The father or the mother whose nerve-gusts are sudden and who cannot reason with children should always keep the rod under lock and key in order that there may be time for the storm to subside before administering it. The wiser parent, who habitually uses reason, may keep it where he pleases.

